# Measuring quality of care at the community level using the contraceptive method information index plus and client reported experience metrics in Bangladesh

**DOI:** 10.7189/jogh.11.07007

**Published:** 2021-03-10

**Authors:** Sharif Hossain, Pooja Sripad, Brady Zieman, Shongkour Roy, Sarah Kennedy, Irfan Hossain, Ben Bellows

**Affiliations:** 1 Population Council, Dhaka, Bangladesh; 2 Population Council, Washington, D.C., USA

## Abstract

**Background:**

Low rates of contraceptive continuation in Bangladesh are a symptom of poor quality family planning (FP) counseling. Improving family planning counseling by the country’s community health care workers (CHWs) could improve contraceptive continuation. This study explores client experiences of care from CHWs, as measured by the method information index plus (MII+) and communication quality metric.

**Methods:**

Conducted in a peri-urban sub-district with low contraceptive use rates, this mixed methods study explores FP client experiences with community-based counseling and referrals by Family Welfare Assistants (FWAs), a CHW cadre providing FP services. Client- and patient-reported experience with community FP services was measured by the MII+ and communication quality metric. A quantitative post-service exit survey was coupled with observations of the interactions between 62 FWAs and 692 female clients to measure FWA and client FP knowledge, FWA capacities, attitudes, quality of FP communication, FP referrals, and contraceptive uptake.

**Results:**

Summary MII+ scores suggest that only 20% of clients reported adequate provision of information for informed decisions. Observations and self-reporting alike suggest moderate to high quality of communication during FWA and client interactions. Despite FWAs’ theoretical knowledge of long-acting reversible and permanent FP methods, few clients were referred to facilities for them; 81% of clients who preferred a pill received it, while only 34% of clients seeking long-acting methods received needed referrals.

**Conclusions:**

Quality community-based FP counseling could help address rising contraceptive discontinuation rates in Bangladesh. While MII and MII+ scores in this study were low, and FWA evinced numerous misconceptions, FWAs demonstrated strong communication skills that facilitate rapport and trust with their clients and communities. Bangladesh’s policy and programs should capitalize upon these relationships and enhance CHWs’ knowledge of all method types, and side effects management, with updated job aids, refresher training, and supervision.

Access to high quality, voluntary family planning (FP) is a right and can improve maternal and newborn health outcomes, advance women's empowerment, and accelerate socio-economic benefits for communities [1,2]. Women with fewer children are more likely to participate in the workplace, increasing economic benefits for their dependents, who become more economically productive themselves [3]. When children are healthier and infant mortality is reduced, the average number of desired children decreases, creating greater demand for contraceptives and strengthening a cycle of continuous improvement in both health and economic development [4-6].

## Contraceptive trends in Bangladesh

Since the 1970s in Bangladesh, primarily due to its effective FP program, sharp declines in fertility have occurred due to increased contraceptive use. The country’s total fertility rate (TFR) declined from 6.3 lifetime births per woman in 1975 to 2.3 in 2017, while the contraceptive prevalence rate (CPR) increased from 8% in 1975 to 62% in 2017 [7]. As contraceptive access and use improved over the past five decades, contraceptive discontinuation actually increased; however, in the most recent Demographic and Health Survey (DHS), more than one third of contraceptive users discontinued their chosen method within one year of its adoption [7]. Bangladesh’s contraceptive discontinuation rates are likely related to the quality of FP counseling and care they received [8-11].

Since the 1970s, Bangladesh’s community health workers (CHWs) have provided primary FP counseling, provision, and referrals [12]. Family Welfare Assistants (FWAs) constitute the country’s CHW cadre with principal responsibility for FP, including counseling, care, and referrals. FWAs also register pregnant women and arrange necessary health care in addition to providing contraceptive pills, condoms, and a second dose of injectable contraception; counseling eligible couples on contraceptive methods and side effects; and referring couples for facility-based contraceptive services, along with other tasks.

## Method information index

Measurement of quality in FP has been an evolving priority for researchers and programs since the 1990 introduction of the Bruce FP quality framework, and is well documented [13-15]. Recent research focuses on utilization of the method information index (MII) as a key measure of FP quality. MII is a validated measure of information retained by a client after FP counseling and is associated with higher rates of contraception continuation for all methods [13,16,17]. Effective counseling helps a client make informed choices about which FP method to select, its use, when to return for further supply, what to expect with respect to side effects, and how to manage them if they occur.

The original MII featured three questions: “Were you informed about other methods?”, “Were you informed about side effects?”, and “Were you told what to do if you experienced side effects?” An updated version of the index, MII+, added the question: “Were you told about the possibility of switching to another method if the method you selected was not suitable?” [18]. An MII score is estimated by the proportion of respondents who answer “yes” to all three questions, and similarly for MII+, the proportion who answer “yes” to all four questions. The MII and MII+ score for all methods is the average of method-specific values, weighted by the proportion of users relying on a particular method.

## Communication quality

The quality of communication between patients and providers of care can affect patient outcomes, including reproductive health (RH) [19]. During contraceptive counseling sessions, interpersonal communication is critical in helping clients select methods appropriate for their RH needs. While the quality of communication at health facilities has been considered, little effort has been made to document and assess this element during community-based health services. A CHW communication quality measure, reflecting experience of care within the client and CHW interaction, is under development [20,21].

Similarly, there are no studies of the association between FP counseling quality in community settings and contraceptive continuation among eligible couples in a country where contraceptive use and discontinuation are both prevalent. This paper presents the baseline findings of an ongoing study measuring client perceptions of their quality of care from FWAs along with their continuation of any contraceptive method for 12 months. This manuscript 1) assesses the validity of MII during community-based FWA counseling by comparing observed vs reported MII, 2) compares observed and reported indices of communication quality, and 3) describes CHWs’ knowledge and practices, including personal beliefs and opinions that may affect the quality of their services.

## METHODS

### Study design

Using mixed methods, this study explored quality of interpersonal communication in community-based FP counseling and referrals. A quantitative post-service survey of women with an unmet contraceptive need investigated the quality of their interactions with FWAs, including the quality of FP counseling (ie, MII+) and communication, FP referrals, and service uptake. A linked survey of FWAs assessed their general knowledge, capacities, and attitudes, in addition to their FP counseling, service provision, and referral practices. We linked these FWA surveys with observations of FWA interactions with clients, including their FP service provision, quality of their counseling, and knowledge, using a checklist.

### Study setting

In collaboration with the Clinical Contraception Service Delivery Programme (CCSDP), Directorate General of Family Planning (DGFP) of Bangladesh’s Ministry of Health and Family Welfare, Keraniganj upazila (eg, sub-district) of Dhaka district was selected due to its proximity to the capital, Dhaka, and relatively low contraceptive use compared to other upazilas. This peri-urban upazila’s 13 Union Health and Family Welfare Centres (UHFWCs) serve a population of 800 000.

### Data collection

Baseline data collection took place from November 2019 to January 2020. Recruitment for the female FP client post-service survey utilized a list of married women who consulted FWAs (from 15 to 20 clients per FWA) for FP information and services and indicated personal unmet FP need. Clients who met the inclusion criteria (eg, non-use of any FP method, or discontinued methods in the last 3 months, and 15 to 35 years of age) were asked if they were willing to participate in a study about their FWA interactions and prospective contraceptive use. Consenting FP clients (n = 949) were interviewed in the local language (Bengali) by a trained data collector using a structured, tested questionnaire.

*Survey of FWAs*: All active FWAs in Keraniganj (n = 62) were interviewed to document their background characteristics, including training, as well as FP knowledge and practices, to better understand both the process and challenges inherent in improving counseling, referral, and care. Factors affecting FWAs’ counseling and referrals were investigated. FWAs were interviewed in the local language by a trained data collector using a structured, tested questionnaire.

*Direct observations:* FWAs’ counseling and services (n = 62) were observed by trained data collectors at clients’ homes, using a checklist. These trained data collectors requested verbal informed consent from all FWAs before quietly observing their provision of services. All observed clients were interviewed upon FWAs’ departures to assess their FWA services.

Ethical approval for this study was provided by the Population Council’s Institutional Review Board (Protocol 874) and Bangladesh Medical Research Council (#20608052019).

### Analysis

Data from FP clients survey, service observations, and the FWA survey were triangulated to assess MII validity, comparing observed vs reported MII, and observed and reported communication skills, as well as estimating FWAs’ knowledge, beliefs, and practices that could influence the quality of their services.

MII was calculated as in previous facility studies, along with the experiential quality of care and referral indicators suggested in the Community Health Worker Performance Measurement Framework [20,22]. MII is calculated by the percentage of women who responded “yes” to all questions in the index. Similarly, experience of care, with communication quality as a proxy indicator, was measured dichotomously by six self-reported items from clients and 10 observed checklist items. A range of indicators for FWAs’ FP knowledge and beliefs are detailed in the FWA survey and triangulated with clients’ self-reported FP service use and referrals. Stata software version 15.0 (Stata Corporation, College Station, TX, USA) was used for statistical analyses.

## RESULTS

### Demographics and FP Counseling Context

The 62 FWAs in this sample were, on average, 46 years old (SD =  ± 10.1). Fewer than half (45%) of these FWAs completed their secondary education, while just more than one quarter (29%) had some amount of higher education and slightly fewer (23%) had a three-year university degree; the remainder did not complete secondary education. A quarter of FWAs (27%) had another occupation prior to becoming an FWA. FWAs reported an average salary of 24 000 BDT (USD 287). Two thirds of FWAs (66%) resided within their assigned service territory (**Table 1**).

**Table 1:** Demographic characteristics and FP counseling context

CHWs characteristics*	n = 62	%
**Age (years):**		
Mean (SD)	46.1	(10.1)
**Education**		
Less secondary	2	3.2
Secondary	28	45.2
Higher secondary	18	29
Bachelors	14	22.6
**Marital status:**		
Currently married	53	85.8
Unmarried	3	4.8
Widowed	6	9.6
**Worked as CHW for over 10 y**	46	77.4
**How long does it take you to get to your village/facility:**		
<15 min	31	50
15-30 min	16	25.8
31-60 min	10	16.1
>60 min	5	7.1
**Monthly salary income in taka**		
Mean (SD)	24241	(4638)
**Clients Characteristics†**	**n = 949**	**%**
**Age (years)‡**		
Mean (SD)	24.5	(5.1)
**Highest education completed:‡**		
No education	51	5.4
Primary	295	31.1
Secondary (incomplete)	524	55.2
Secondary and above	79	8.3
**Parity‡**		
None	37	4.0
1	320	33.7
2	399	42.0
3 and more	193	20.3
Mean (SD)	(0.95)	1.85
**Family planning user type:‡**		
Past user	774	81.6
New user	175	18.4

FP – family planning, CWH – community health worker, SD – standard deviation

*CHW survey.

†Client survey.

‡Self-reported.

The 949 clients surveyed were, on average, 25 years old (SD = ± 5.1). Most FP clients had not completed their secondary education (55%), and nearly one third (31%) had not completed primary school. On average, FP clients had been pregnant twice before this survey. Almost all clients’ husbands were either cohabitating or visited their wives at least four days a week. Eighty-two percent of these clients reported using a FP method in the past prior to the 3 months preceding the interview.

### FP Information provided: observed counseling vs self-reported MII and MII+

Women who met eligibility criteria and received a method were included in this analysis (n = 697). More than two thirds of clients were provided accurate method-specific information to support an informed choice (eg, how to use a method, its effectiveness, advantages and disadvantages, when to return to a facility). Only two-fifths of clients were given accurate information on side effects, and only 5% of clients received accurate information on danger signs for their preferred method (**Table 2**).

**Table 2:** Information provided observed vs self-reported MII

Observed MII*	N = 697	%
1. Provider gave accurate information about method use	482	69
2. Provider gave accurate information about method effectiveness	472	68
3. Provider gave accurate information about method advantages/disadvantages	476	68
4. Provider gave accurate information about method side effects	273	39
5. Provider gave accurate information about when to return to the facility	434	62
6. Provider gave accurate information about danger signs when using the preferred method	37	5
***Summary observed method information score – mean (SD)***	3.12 (1.89)	
**Self-reported MII†**	**N = 697**	**%**
1. During your last visit/meeting, were you told about other methods of FP that you could use (methods other than the one you received)?	505	73
2. At that time, were you told about side effects or problems you might have with the method?	254	36
3. Were you told what to do if you experienced side effects or problems? ‡	173	68
4. Were you told that you could change method or switch to another method if you have any issue with the method you just received?	443	64
**Yes, to all three questions above (1 + 2 + 3)**	**151**	**22**
**Yes, to all four questions above (1 + 2 + 3+4)**	**141**	**20**
5. For the method you have just accepted, were you told how to use your method?	542	78
6. Did the FWA talk about warning signs associated with the method you selected?	23	3
7. How satisfied were you with the services you received from the FWA?	315	40
***Summary MII Score (1 + 2 + 3) – mean (SD)***	1.33 (1.05)	
***Summary MII+Score (1 + 2 + 3+4) – mean (SD)***	1.97 (1.37)	

MII – method information index, SD – standard deviation, FWA – family welfare assistant

*Observation of service provision.

†Client survey.

‡Sample size (N = 254).

Women who received a method were asked to report whether they were informed about alternative contraceptive methods, side effects management, and method switching (ie, MII+). Women who respond “yes” to all four questions are considered to have received full information based on MII+. **Table 2** shows that 73% of women reported adequate information on “how to use the method she just received,” 36% recalled “side effects or problems she might have with the method,” 68% mentioned “what to do if she experiences side effects,” and 64% were informed about method switching. Only 20% of clients reported that information in all four questions was offered to help their informed choice, while slightly more, 22%, reported receiving information included in three questions (**Table 2**).

Although the questions differed somewhat, there is a high degree of positive correlation between observed (6 items) and self-reported data (4 items) for the method information indices (rho = 0.601). Around two thirds of clients received adequate information during FP services to aid their informed choice, while one third lacked adequate information (data not shown, based on summary scores in **Table 2**).

### Observed v. self-reported communication quality

Observations and self-reports alike suggest moderate to high communication quality during FWA and client interactions. The observations reflect lower communication quality and experience of care than self-reporting in some cases, with similar levels in others, as seen in analogous measures. Although the observations demonstrate that FWAs greeted their clients in 81% of cases, almost all clients reported being greeting by their FWA. Observations and the client survey do reveal similar proportions of FWAs introducing themselves to clients ( ~ 90%), explaining the purpose of their visit ( ~ 67%), providing information in simple words or manner understandable to clients, including using the local dialect (99%), encouraging clients to ask questions (42% to 45%), and answering questions clearly (97%). Excluding language sensitivity, few FWAs were found to use examples (17%) and hardly any used visual aids (3%). Almost all (97%) observations and self-reporting suggest FWAs are amenable to answering questions by clients, and some observations describe careful and active listening by FWAs.

Although the questions differed somewhat, there is a high degree of positive correlation between observed (10 items) and self-reported data (6 items) for the communication quality indices (rho = 0.656). Around two thirds of clients had quality communications with their FP service providers, while one third experienced poor communications (data not shown, based on summary scores in **Table 3**).

**Table 3:** Observed vs self-reported communications

Self-reported communication items*	N = 949	%	Observed communication items†	N = 949	%
Did the FWA greet you in a friendly way?	935	99	Did the FWA greet the client?	769	81
Did the FWA introduce her/himself? (if 1st meeting)	836	88	Did the FWA introduce herself?	876	92
Did the FWA explain the purpose of the visit?	644	68	Did the FWA explain or ask the purpose of visit?	623	66
Was the information given to you by the FWA easy to understand?	943	99	Did the FWA use simple words in local language?	942	99
			Did the FWA demonstrate, give examples, and/or use visual aids (as appropriate) while communicating	163	17
			Did the FWA use visual aids?	25	3
Did you ask the FWA questions?	394	42	Did the FWA encourage the client if she has any questions?	425	45
			Did the FWA listen and watch carefully when the client talks?	906	95
			Did the FWA use appropriate ‘non-verbal’ body language (check for nodding, eye contact, leaning forward or backwards, facial expressions etc.)?	904	95
Did the FWA answer your questions clearly?	385	97	Did the FWA answer questions simply?	459	97
***Summary score – mean (SD)***	3.62 (1.27)		***Summary score – mean (SD)***	6.42 (1.78)	

FWA – family welfare assistant, SD – standard deviation

*Client survey.

†Observation of service provision.

### FWA knowledge, beliefs, and practices

**Figure 1:**
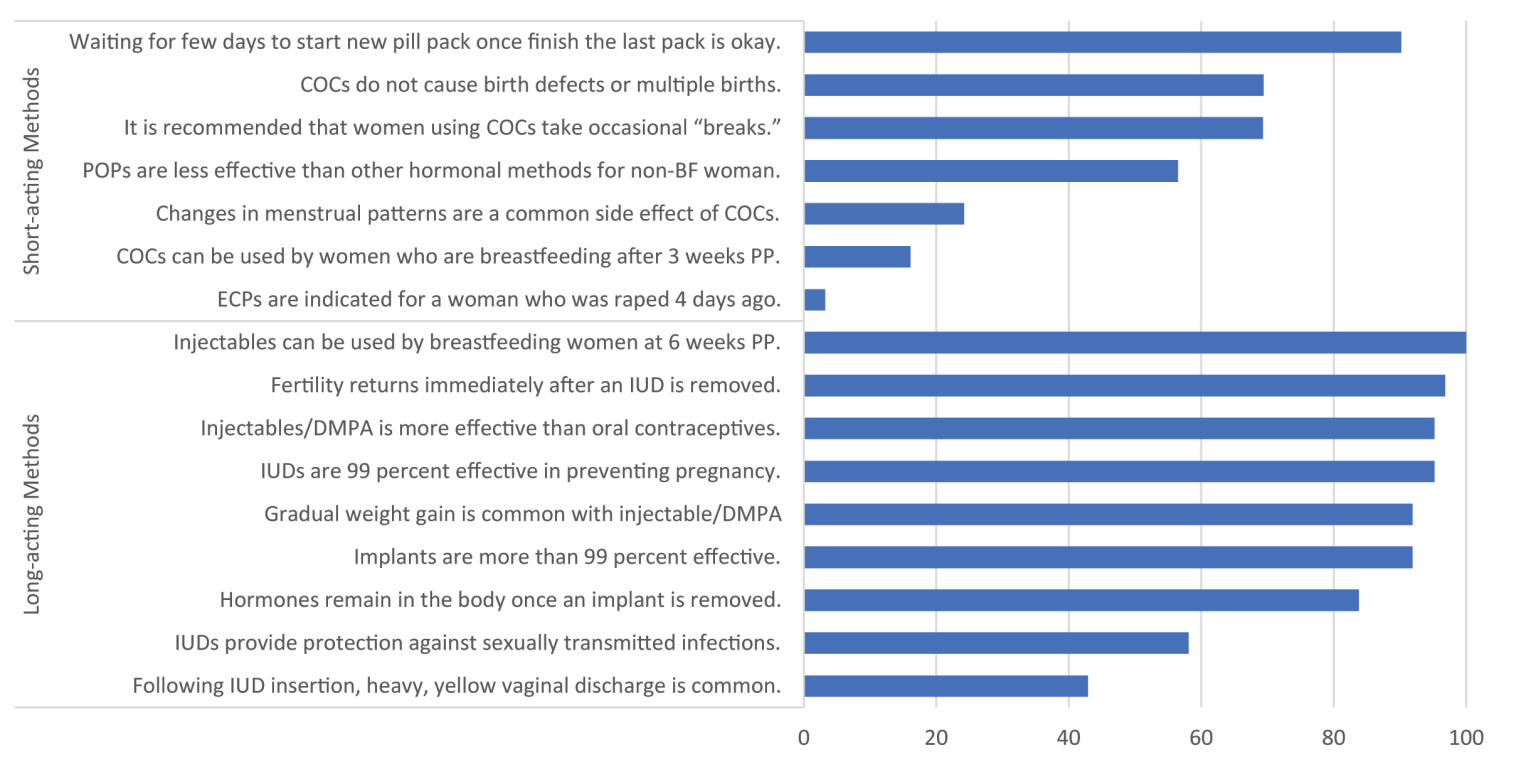
Percentage of FWAs on knowledge and beliefs about FP methods (n = 62). FWA – family welfare assistant, FP – family planning, COC – combine oral contraceptive, POP – progesterone only pill, BF – breast feeding, PP – post-partum, ECP – emergency contraceptive pill, IUD – intrauterine device, DMPA – depot-medroxyprogesterone acetate.

The FWA survey revealed diverse knowledge and beliefs relating to short- and long-acting FP methods (**Figure 1**). FWAs offered assessment, counseling, and provision of short-acting methods, with counseling and referrals for long-acting methods, yet FWAs have poor knowledge of short-acting as well as long-acting methods. About 90% of FWAs reported that “it is okay for a woman to wait a few days to start a new pack of pills once she finishes the last pack.” Almost all FWAs thought that “fertility returns immediately after an IUD is removed,” and about 60% of FWAs reported that “IUDs provide protection against sexually transmitted infections” (**Figure 1**).

**Figure 2:**
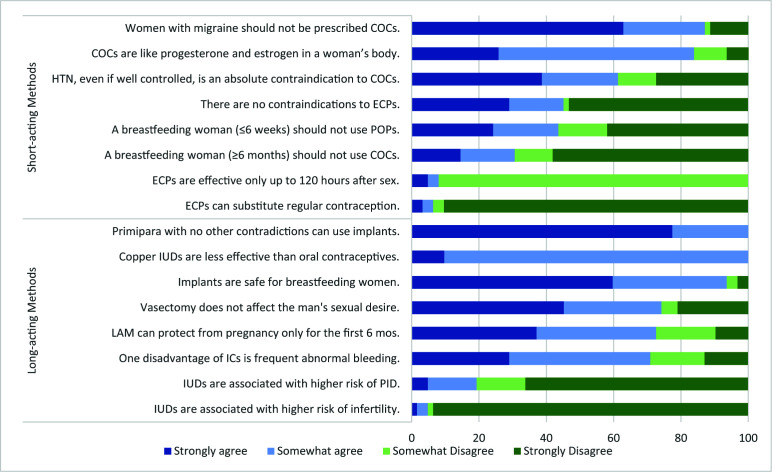
Percent of FWAs who agree/disagree with statements about FP methods (n = 62). FWA – family welfare assistant, FP – family planning, COC – combine oral contraceptive, HTN – hypertension, POP – progesterone only pill, ECP – emergency contraceptive pill, IUD – intrauterine device, LAM – lactational amenorrhea method, IC – injectable contraceptives, PID – pelvic inflammatory diseases.

When asked to agree or disagree with general statements about FP methods, FWAs revealed several misconceptions about short- and long-acting, reversible and permanent, methods **(****Figure 1** and **Figure 2****)**. Approximately 70% of FWAs thought it was recommended “a woman should occasionally take a break from COCs.” Similarly, about 90% of FWAs disagreed or strongly disagreed with the statement “ECPs can be substituted for regular contraception.” Statements about long-acting, reversible and permanent methods also manifested in misinformed responses. All FWAs agreed or strongly agreed with the statement “Copper IUDs are less effective than oral contraceptives,” and about 25% disagreed or strongly disagreed that “vasectomy does not affect the man’s sexual desire” (**Figure 2**).

Despite FWAs’ theoretical knowledge of long-acting, reversible and permanent, methods, few clients were referred to facilities for those methods (**Table 4**). During recent interactions with FWAs, more than a quarter of women (27%) reported receiving information only (eg, handouts or explanation) from an FWA, while three quarters (73%) reported receiving either a method or referral (**Table 4**). Seventy-nine percent of observed clients expressed a method preference with FWAs, while the remaining 21% of clients either didn’t express a preference or method preference was not discussed (data not shown). Forty-seven percent of observed clients were provided their preferred method (pill or condom), while 18% of observed clients were provided with a non-preferential method. Eight percent of observed clients were referred for either preferred or other methods (**Table 4**).

**Table 4:** Information, services, and referrals provided by FWAs: direct observations*

	n = 949	%
**Visit outcomes**		
Information only	252	26.6
Received preferred method of pill and condom	448	47.2
Received referral for method of IUD, injectable, implant, and female sterilization (preferred)	54	5.7
Received referral for method of IUD, injectable, and implant (non-preferred)	20	2.1
Received other non-preferred modern method	175	18.4

FWA – family welfare assistant, IUD – intrauterine device

*Observation of service provision.

## DISCUSSION

Quality health care is a right, and counseling quality is a critically important dimension of any FP program [23]. Counseling requires dual communication between service providers and clients in which providers offer enough information for each client’s realization of informed choice. Quality counseling ensures every client has the information necessary to manifest her or his own RH goals.

The baseline FP counseling measured by standard MII and MII+ in this study was of low quality, whereas the novel communication quality index score was moderate-to-high. While poor FP counseling quality has negative implications for sustained, voluntary contraceptive continuation, good communications during FWA and client interactions suggest positive experiences of care. Further research with this cohort will continue, but these initial findings suggest that to sustain broad, equitable gains in FP, particularly in low- and middle-income country (LMIC) settings where contraceptive prevalence is already mid-range (eg, 40%-60%), quality of care must be a primary focus, especially among disadvantaged clients.

### Validating community health metrics

This study demonstrates that the quality of FP care can be measured during its provision in community settings. These measures were deployed and tested as a part of the Frontline Health project, which seeks to standardize and offer transferrable measures of CHW performance and utility within community health systems [20]. Although MII and MII+ have been validated in clinical studies and assessed in household surveys, this is the first study to validate it in community level FP service delivery [16-18,24]. Reporting MII+ in community-level service provision is useful for monitoring any community FP program’s progress. Communication quality during CHW and client interactions, meanwhile, captures aspects of clients’ experiences of care without indicating specific health outcomes.

This study validated the MII, MII+, and communication quality metrics, with its self-reported and observed measures closely correlated. Despite the positively skewed self-reporting of some communication quality elements, this study, like other validation efforts linking subject observation and client self-reporting, shows promising results with implications for special studies and routine program monitoring [25-27]. Self-reported measures are an important data source for program managers seeking to improve efficiency and reduce costs.

Method-specific and overall MII values can range from 0 to 100; a lower MII value reflects less information retained by the client. In two previous studies, MII and MII+ values were much higher. The same studies also noted that MII values were predictive of higher rates of all-method contraceptive continuation [18,28]. Similar to a recent study of Performance Monitoring and Accountability (PMA) data in Ethiopia [29], only 22% and 20% of this study’s respondents had high MII and MII+ scores, respectively, indicating that few women recalled receiving adequate information from CHWs. By contrast, in a study of respondents at urban franchised clinics in Pakistan and Uganda, 65% and 73% of women, respectively, recalled receiving information with all three MII elements [21]. Given the significant barriers to facility-based services, community service delivery can improve access to FP services, but quality may be low, as this study demonstrates. These results indicate that Bangladesh’s overall progress in increasing modern contraceptive use has not been complemented by corresponding increases in FP counseling quality.

Quality communication conceptually coheres, and may be positively correlated with, trust in CHWs as well as empowered clients’ agency in seeking FP services [30,31]. In a 1997 study of quality of care and contraceptive use in rural Bangladesh, high quality of care was associated with 72% greater likelihood of continued use of any contraceptive method. The same study also found higher standards of care were associated with a 27% increase in subsequent adoption of contraception by non-users [32]. Measures of the experience of care such as communication quality offer insights into both the significance and acceptability of community-based care. This study’s self-reported communication quality score was comparable to a score of communication quality during community-based postnatal care services in Kenya [21]. This study reinforces that CHWs have positive relationships with others in their communities, likely due to their associations and socio-cultural similarity. These invaluable elements can be capitalized upon, if complemented by improved FP knowledge among CHWs and their clients.

### Enhance CHW capacity for quality FP counseling

FWAs’ knowledge, beliefs, and practices may be constraining clients’ quality of care, including access to FP methods. Low quality counseling could reflect knowledge deficiencies among FWAs due to inadequate FP training and practice. Most FWAs have job aids during home visits, but comprehensive training and FP refresher training are not standard, and most FWAs never receive any follow up FP training after their initial job training. Sporadic training on topics such as child and adolescent nutrition, newborn care, pregnancy, and immunization is provided, but not for FP.

During this study more women received short-acting than long-acting methods (including referrals), which may be due to judgments by FWAs about method suitability for individual women, women’s method preferences, method availability, or could reflect FWA favorable bias toward certain methods (eg, pills). FWA decisions for promoting certain methods and referrals may be tied to wider health system shortages and the availability of trained providers and methods at facilities, particularly long-acting reversble contraception (LARCs) and permanent methods. In Bangladesh, only one quarter of health facilities provide LARCs or permanent methods, while few provide male or female sterilization services [33].

Our findings suggest a need to not only improve measurement of FP counseling quality but strengthen CHWs’ knowledge of comprehensive FP methods, and where to procure them if supply is eclipsed by demand. While Ruth Simmons et al. (1988) perceived FWAs as having “beyond-supply” social effects, since then the FWA cadre has failed to scale their capacity to deliver FP [34]. Managing FP program quality is a perennial challenge for program administrators and policymakers. When considering quality improvement mechanisms, both generally and within community health programs, program administrators and policymakers need reliable metrics like the MII+ to benchmark progress [35].

These findings suggest that FWAs need to be sufficiently trained in FP information, client segmentation, and referral, and routinely provided with updated, accurate information, while adequately incentivized to spend more time in household visits, supported by sufficient monitoring and supervision. Because FWAs have been working for decades in community health provision in Bangladesh, with compensation unequal to other community-based cadres (eg, Health Assistants), they may be unmotivated and reluctant to revise their FP counseling approaches [36]. Further exploration of FWA motivation, using validated measures in relation to their practices, could be helpful [37].

## CONCLUSIONS

Performance measurement and monitoring of community health care services remains one of the weakest components of health systems in many countries. Client-reported experience measures of community FP services can be captured through MII, MII+, and communication quality indices during community service provision. While MII scores were low in this study, and FP CHWs (FWAs) expressed many misconceptions about FP, FWAs also demonstrated strong communication skills that build rapport and trust with their clients.

Bangladesh’s health policymakers should aim to enhance CHWs’ knowledge of FP methods and side effects management, and reinforce their counseling and communication skills, refresher training, and improved supervision, ultimately strengthening positive CHW and client relationships, improving FP counseling quality, and addressing the country’s high rates of contraceptive discontinuation. Implementation research in Bangladesh and elsewhere should embed routine, community-based client-reported experience measures (eg, client-reported quality metrics) for effective community health service monitoring, program management, and policy reforms.
